# Obesity and metabolic comorbidity in bipolar disorder: do patients on lithium comprise a subgroup? A naturalistic study

**DOI:** 10.1186/s12888-021-03572-w

**Published:** 2021-11-10

**Authors:** Jake Prillo, Jocelyn Fotso Soh, Haley Park, Serge Beaulieu, Outi Linnaranta, Soham Rej

**Affiliations:** 1 GeriPARTy Group, Division of Geriatric Psychiatry, Jewish General Hospital, McGill University, Montreal, Canada; 2 Douglas Mental Health University Institute and Department of Psychiatry, McGill University, Montreal, Canada

**Keywords:** Obesity, Metabolic syndrome, lithium, Insulin resistance, Bipolar disorder

## Abstract

**Background:**

Bipolar disorders (BD) are associated with increased prevalence of obesity and metabolic syndrome (MetS). Nevertheless, there is a wide range in prevalence estimates, with little known about the contributions of pharmacotherapy. It has been suggested that lithium might have a more favorable metabolic profile. We hypothesized that lithium use is associated with less increased body mass index (BMI), MetS, and type II diabetes, when compared with non-lithium users (those on anticonvulsants, second-generation antipsychotics).

**Methods:**

Cross-sectional study of 129 patients aged 18–85 with bipolar disorder, followed at tertiary care clinics in Montreal. Patients using lithium were compared with those not on lithium, for body mass index and metabolic syndrome.

**Results:**

The prevalence of obesity and metabolic syndrome in the sample of lithium-using patients with BD was 42.4 and 35.7% respectively, with an average BMI of 29.10 (+/− 6.70). Lithium and non-lithium groups did not differ in BMI or prevalence of MetS. However, compared to the non-lithium group, lithium users had lower hemoglobin A1C (5.24 +/− 0.53 versus 6.01 +/− 1.83, U = 753.5, *p* = 0.006) and lower triglycerides (1.46 +/− 0.88 versus 2.01 +/− 1.25, U = 947, *p* = 0.020).

**Conclusions:**

There is a high prevalence of obesity and metabolic syndrome among patients with bipolar disorder. However, this did not appear to be associated with lithium use, when compared to those not on lithium. The lithium subgroup was also associated with lower prevalence of type II diabetes. Future prospective and intervention studies with larger sample sizes are necessary to further explore the association between lithium and insulin resistance, as well as its underlying mechanisms.

## Introduction

Lithium remains a first-line therapy in BD, with indications in acute mania, depression, and maintenance treatment [1]. Additionally, it is associated with unique anti-suicidal properties [2]. Despite this, lithium is often avoided for its adverse physical effects including reduced renal function, hypothyroidism, hyperparathyroidism and weight gain [3].

Among these adverse physical effects, weight gain is especially concerning, as it can lead to obesity. This is a constituent of MetS, a constellation of risk factors notably associated with cardiovascular disease and stroke [4]. Weight gain is further recognized in the literature as contributing to poor physical health and early mortality [5], treatment non-adherence [6], worsening psychiatric outcomes [7], and elevated economic costs [8]. Mitigating obesity is highlighted in numerous consensus guidelines [1, 9], which state that body mass index, type II diabetes, and dyslipidemia be assessed at treatment baseline and then regular intervals thereafter.

One question that arises is whether lithium treatment in patients with BD confers a more favourable metabolic profile, compared to other treatments (anticonvulsants, second-generation antipsychotics). While it is known that patients with BD are at an increased risk of obesity [10], the prevalence rate remains heterogeneous with estimates ranging between 20 and 65% of patients with BD [11]. The same is true for MetS, including increased waist circumference, type II diabetes, and dyslipidemia. Further, the relative contributions of the bipolar illness itself, aging, and mood-stabilizing medications need to be better clarified. A recent large-scale meta-analysis has shown second-generation antipsychotics to be associated with obesity, cardiovascular disease, and premature death [12]. On the other hand, a number of randomized clinical trials (RCTs) and cohort studies suggest that lithium causes less weight gain, especially compared to olanzapine and quetiapine [13–17]. Lithium is also associated with a lower risk of stroke [18]. Yet in these studies, BMI and metabolic effects are not reported as primary outcomes. These trials also include strict ‘treatment arms’ that do not reflect true clinical prescribing patterns (for example, medication combinations, augmentation, and polypharmacy).

The purpose of this study is to 1) estimate the prevalence of obesity and MetS amongst a cross-sectional sample of patients with BD. We will also 2) examine the prevalence of constituents of MetS, including BMI, waist circumference, hypertension, lipid profile, and diabetic screening tests. Finally, we will 3) assess whether there are any differences between lithium and non-lithium patients with BD. In this paper, we hypothesize that lithium users will have less medical comorbidity, including obesity, MetS, and diabetes.

## Materials and methods

We combined data from two studies that examined physical comorbidity in bipolar patients. These studies were chosen for secondary analysis, as they were readily available to our research team. We have provided individual descriptions of the methods of each study below. Specific inclusion or exclusion criteria of the studies have been previously reported [19, 20].

Study 1 assessed atorvastatin (20 mg/day) versus placebo in the treatment of lithium users with nephrogenic diabetes insipidus (DI). It was a double blind, placebo-controlled RCT at three tertiary mental health sites in Montreal, Québec: Douglas Mental Health University Institute (DMHUI), Jewish General Hospital (JGH), and McGill University Health Centre (MUHC). Patients were lithium users aged 18–85, with any psychiatric disorder. At baseline, patients self reported demographic information, past medical and psychiatric history, and somatic complaints. Current medication was obtained from medical charts and confirmed by the patient. At each visit (baseline, 4-weeks, 12-weeks), psychiatric symptoms were measured using the Montgomery-Asberg Depression Scale (MADRS) [21] and Young Mania Rating Scale (YMRS) [22]. Finally, physical measures (BMI and waist circumference), serum cholesterol levels, serum glucose levels, and thyroid function were also measured at each visit. For this analysis, baseline data from Study 1 was used from patients with BD (*n = 43*).

Study 2 examined the relationship between mood, sleep, and food intake in bipolar patients. Patients were recruited from those presenting at the Bipolar Disorders clinic at the DMHUI. This is a specialized clinic where the diagnosis of patients has been systematically evaluated and scrutinized before acceptance. Patients were 18 years or older and were receiving care exclusively as outpatients. Medication, demographic, clinical, and laboratory information were retrieved for the initial visit. Two in-person visits, 2 weeks apart, were used to evaluate physical measures and mood. For this analysis, baseline data from Study 2 was used (*n = 86*).

### Exposures – Lithium users and non-users

Primarily, we classified patients with BD into current lithium users or non-users. This was achieved by a chart review of any prescriptions within a 2-week period before or after the initial baseline visit. Psychotropic exposure categories were dichotomous, without consideration of treatment dose or duration.

### Primary outcome

Our primary outcome was obesity, measured via body mass index (BMI). Obesity was chosen as lithium and other mood stabilizers have frequently been associated with weight gain, which may lead to treatment non-adherence and metabolic complications. It is also a value that can be easily measured and trended, no matter the healthcare setting. BMI was calculated using a standard formula: BMI = weight (kg) / height^2^ (meters). A BMI ≥ 30 was taken to indicate obesity, according to the National Institute of Health (NIH).

### Secondary outcomes

Our secondary outcomes were average BMI, BMI classes, MetS, and hemoglobin A1c (HbA1c). The BMI classes are designated by the NIH: BMI < 19.0 (underweight), BMI 19–24.9 (normal), BMI 25.0–29.9 (overweight), BMI 30.0–34.9 (class I obesity), BMI 35.0+ (class II & III obesity).

MetS was diagnosed in patients who fulfilled three or more of the five criteria based on the National Cholesterol Education Program Treatment Panel III (NCEP III): 1) Abdominal obesity: waist circumference ≥ 102 cm for men and ≥ 88 cm for women. Waist circumference was measured in two ways: at the top of the iliac crest as per the NIH, and midway between the last palpable rib and the top of the iliac crest according to World Health Organization (WHO) standards; 2) Glucose dysmetabolism: impaired fasting plasma glucose (5.5–6.9 mmol/L) or diabetes (fasting plasma glucose ≥7 mmol/L 3) Dyslipidemia: elevated plasma triglyceride (≥1.7 mmol/L) 4) Dyslipidemia (second, separate criteria): decreased high-density lipoprotein (HDL) (< 1.03 mmol/L for men and < 1.30 mmol/L for women); and 5) Hypertension. Hypertension was defined as a high blood pressure reported on history or necessitating pharmacologic intervention according to a medication report.

Hemoglobin A1c was measured to assess glucose dysmetabolism and examine the prevalence of type II diabetes. Hemoglobin A1c is the most widely used clinical test to diagnose diabetes, indicating the mean glucose concentration over 120 days, though it is not traditionally used in the diagnosis of MetS.

### Exploratory outcomes

Finally, our exploratory outcomes were the constituents of MetS: abdominal obesity, glucose dysmetabolism, dyslipidemia, and hypertension. Additionally, we supplemented these with thyroid function, as it is a frequent adverse event during lithium use. Hypothyroidism was characterized as a thyroid stimulating hormone (TSH) value > 5.0mIU/L or necessitating thyroid hormone replacement.

### Statistical analysis

Data was initially assessed for normality using the Shapiro-Wilk test. We compared the exposure groups for demographic values, as well as for primary, secondary, and exploratory outcomes. We used Mann-Whitney U tests to examine continuous variables (eg. HbA1C) for non-normal distributions. Chi-squared tests were employed for dichotomous variables (eg. Diabetes Yes/No). An additional exploratory ANCOVA analysis was performed to assess for the association of lithium and BMI after controlling for concomitant antipsychotic use, as well as age and gender. A two-tailed alpha of 0.05 was used to determine statistical significance and all analyses were performed using IBM SPSS 26.0 (IBM SPSS Inc., Chicago, IL, USA).

## Results

In total, 129 patients with BD were included in the analysis of this study. Of these, 66 were lithium users and 63 were non-users. The lithium group was 43.9% male, and the mean age was 49.1 (±11.78). The majority (*n* = 50, 76.9%) of lithium users had an age of onset of psychiatric symptoms < 30 years of age, with on average more than four mood episodes in the course of their illness.

Table 1 summarizes the study participants’ baseline demographic and clinical characteristics. Ultimately, the lithium and non-lithium groups differed significantly in their age of onset (*p* = 0.017, x^2^ = 13.8), number of mood episodes (*p* = 0.001, x^2^ = 15.9), and their baseline MADRS (4.30 ± 4.62 versus 6.81 ± 6.93 respectively) and YMRS scores (4.42 ± 8.03 versus 15.90 ± 12.80 respectively). There were higher standardized scores for depression and mania in the non-lithium group. Additionally, there were also significant differences in the patients’ medication profiles. Intuitively, a higher proportion of non-lithium users were on antipsychotic medications and anticonvulsants, as well as antidepressants. More specifically, 76.2% of non-lithium users were on antipsychotics, compared to only 58.7% of lithium users (*p* = 0.036, x^2^ = 4.375). This pattern continued for anticonvulsants (82.5% of non-lithium users, versus 36.5%) and antidepressants (57.1% for non-lithium users, versus 38.1%). In terms of somatic medications, the non-lithium group was also on more medications for diabetes and dyslipidemia.

**Table 1:** Demographics & Clinical Characteristics in Lithium Users and Non-Users with Bipolar Disorder (BD), *n* = 129

Variable	Li Users (***n*** = 66)	Non-Li Users (***n*** = 63)	Stats
**Age (years),** [mean (±SD)]	49.05 (11.78)	46.71 (11.20)	U = 1850.5, *p* = 0.281, z = −1.077
**% Male,** [% (*n*=)]	43.94% (*n* = 29)	53.96% (*n* = 34)	*p* = 0.043, x^2^ = 0.169
**Age of onset of any psychiatric symptoms,** [% (*n*=)]			*p* = 0.017, x^2^ = 13.8
Age < 18	33.84% (*n* = 22)	59.68% (*n* = 37)	
Age 18–30	43.08% (*n* = 28)	24.19% (*n* = 15)	
Age 30–50	18.46% (*n* = 12)	12.90% (*n* = 8)	
Age > 50	4.62% (*n* = 3)	0.0% (*n* = 0)	
Not known	0.0% (*n* = 0)	3.23% (*n* = 2)	
**Total number of previous mood episodes,** [% (*n*=)]			*p* = 0.001, x^2^ = 15.9
1 episode	11.67% (*n* = 7)	0% (*n* = 0)	
2 episodes	10% (*n* = 6)	0% (*n* = 0)	
3 episodes	8.33% (*n* = 5)	3.33% (*n* = 2)	
> 4 episodes	70% (*n* = 45)	91.67% (*n* = 55)	
**MADRS score,** [mean (±SD)]	4.30 (4.62)	6.81 (6.93)	U = 4922.5, *p* = 0.045, z = −2.01
**YMRS score,** [mean (±SD)]	4.42 (8.03)	15.90 (12.80)	U = 719, *p* = 1.57^-12, z = −7.07
**Number of psychotropic medications,** *n* = 126**,** [mean (±SD)]	3.13 (1.72)	3.40 (1.40)	U = 1738, *p* = 0.280, z = −1.080
On anticonvulsants, [n (%)]	36.5% (*n* = 23)	82.5% (*n* = 52)	*p* = 1.414 × 10–7, x^2^ = 27.70
On antidepressants, [n (%)]	38.1% (*n* = 24)	57.1% (*n* = 36)	*p* = 0.032, x^2^ = 4.582
On antipsychotics, [n (%)]	58.7% (*n* = 37)	76.2% (*n* = 48)	*p =* 0.036, x^2^ = 4.375
**Number of non-psychotropic medications,** [mean (±SD)]	0.67 (1.004)	2.37 (2.951)	U = 674, *p =* 0.001, z = −3.371
For hypertension, *n* = 117, [n (%)]	13.0% (*n* = 7)	19% (*n* = 12)	*p* = 0.791, x^2^ = 0.374
For diabetes, *n* = 117, [n (%)]	7.4% (*n* = 4)	22.2% (*n* = 14)	*p* = 0.027, x^2^ = 4.902
For dyslipidemia, *n* = 117, [n (%)]	3.7% (*n* = 2)	22.2% (*n* = 14)	*p* = 0.004, x^2^ = 8.447
For thyroid, *n* = 105, [n (%)]	18.4% (*n* = 9)	23.2% (*n* = 13)	*p* = 0.543, x^2^ = 0.371

*Abbreviations***:** *Li* Lithium, *Stats* Statistics, *MADRS* Montgomery-Asberg Depression Rating Scale, *YMRS* Young Mania Rating Scale

Table 2 summarizes obesity, BMI, and other physical health outcome data. With respect to primary outcome, 42.42% of lithium-using and 34.92% of non-lithium bipolar patients had obesity. There was no statistically significant difference in BMI between the lithium and non-lithium groups (*p* = 0.384, x^2^ = 0.758). Similarly, lithium users and non-users did not significantly differ in regards to BMI, after adjusting for age, gender, and antipsychotic use.

**Table 2:** Body Mass Index (BMI) and Physical Health Outcomes in Lithium Users and Non-Users with Bipolar Disorder (BD), *n* = 129

Variable	Li Users *(n =* 66*)*	Non-Li Users *(n =* 63*)*	Stats
**Obesity, [n(%)]**	42.42% (*n =* 28)	34.92% (*n =* 22)	*p* = 0.384, x^2^ = 0.758
**Mean BMI,** (kg/m^2^) [mean (±SD)]	29.10 (+/−6.70)	30.20 (+/−8.57)	U = 2051, *p* = 0.895, z = −0.132
**BMI stratification,** [n (%)]			*p* = 0.184, x^2^ = 4.85
Underweight, < 19	0% (*n* = 0)	0, 0	
Normal, 19–25	30.30% (*n =* 20,)	33.33%, (*n =* 21)	
Overweight, 25–30	27.27% (*n =* 18)	31.75%, (*n =* 20)	
Class I Obesity, 30–35	25.76%, (*n =* 17)	11.11%, (*n =* 7)	
Class II & III Obesity, > 35	16.67%, (*n =* 11)	23.81%, (*n =* 15)	
**Metabolic Syndrome** [n (%)]	35.71% (*n* = 15)	44.18% (*n* = 19)	*p* = 0.623, x^2^ = 0.24
**Waist circumference, NIH**			
Male, [mean (±SD)]	107.91 (20.51)	107.77 (16.79)	U = 1906.5, *p* = 0.416, z = −0.813
Female, [mean (±SD)]	98.85 (16.28)	105.97 (19.03)	
**Glucose Metabolism**			
Fasting Blood Glucose (mmol/L), *n = 107* [mean (±SD)]	5.52 (1.03)	6.10 (2.90)	U = 1344.5, *p* = 0.712, z = −0.369
HbA1C (mmol/L), *n = 95* [mean (±SD)]	5.24 (0.53)	6.01 (1.83)	U = 753.5, ***p*** = 0.006*, z = −2.76
Diabetes, *n* = 87, [n (%)]	16.67% (*n* = 7)	33.33% (*n* = 15)	*p* = 0.088, x^2^ = 3.19
**Lipid Profile**			
Total Cholesterol (mmol/L), *n = 103* [mean (±SD)]	4.72 (1.02)	4.92 (1.10)	U = 1233, *p* = 0.582, z = −0.55
HDL (mmol/L), *n = 102*, [mean (±SD)]	1.30 (0.33)	1.24 (0.37)	U = 1084.5, *p* = 0.162, z = −1.40
LDL (mmol/L), *n = 100*, [mean (±SD)]	2.79 (0.87)	2.75 (0.89)	U = 1110, *p* = 0.377, z = −0.883
Triglycerides (mmol/L), *n = 102*, [mean (±SD)]	1.46 (0.88)	2.01 (1.25)	U = 947, ***p*** = 0.020*, z = −2.32
Dyslipidemia, *n* = 96, [n (%)]	50%, (*n* = 23)	70%, (*n* = 35)	*p* = 0.06, x^2^ = 4.007
**Hypertension**			
Hypertension, *n* = 117, [n (%)]	12.96%, (*n* = 7)	19.05%, (*n* = 12)	*p* = 0.455, x^2^ = 0.791
**Thyroid Function**			
TSH, *n = 103*, [mean (±SD)]	2.39 (1.26)	2.60 (1.43)	U = 1187, *p* = 0.411, z = −0.823
Hypothyroidism, *n =* 102, [n (%)]	41.81%, (*n* = 23)	59.57%, (*n* = 28)	*p* = 0.112, x^2^ = 3.196

*Complete information was available on all participants for BMI and waist circumference. For other domains, the number of patients available is indicated next to the name of each variable (n=).*

*Abbreviations***:** *Li* Lithium, *Stats* Statistics, *BMI* Body mass index, *HbA1c* Hemoglobin A1c, *HDL* High-density lipoprotein, *LDL* Low-density lipoprotein, *TSH* Thyroid stimulating hormone

* = *statistical significance at two-tailed p < 0.05*

For secondary outcomes, mean BMIs were on the cusp of overweight and class I obesity; mean BMI of the lithium-using bipolar patients was 29.10 (± 6.70) kg/m^2,^ and non-lithium users 30.20 (+/− 8.57) kg/m^2^. There was no statistically significant difference in BMI between the two groups. The vast majority of the 66 lithium-using patients were outside of the normal BMI range: 27.3% overweight, 25.8% class I obesity, and 16.7% class II & III obesity. The prevalence of MetS in lithium users was similarly elevated at 35.7%. There was no significant difference between lithium users and non-lithium users. However, there was a statistically significant difference in glucose metabolism as measured by HbA1C. Lithium use was associated with lower HbA1c overall (5.24 ± 0.53 versus 6.01 ± 1.83, U = 753.5, *p* = 0.006).

Lastly, for exploratory outcomes, lithium usage was associated with lower triglyceride levels than non-lithium users (1.46 ± 0.88 versus 2.01 ± 1.25, U = 947, *p* = 0.020). There was no difference in the remaining constituents of MetS, including waist circumference, fasting plasma glucose or diabetes, cholesterol profile or dyslipidemia, or hypertension. There was also no difference in thyroid function, despite lithium’s well-established association with hypothyroidism.

## Discussion

We present a sample of patients with BD in a tertiary care, naturalistic setting, where polypharmacy is common. Overall, the prevalence of obesity and MetS in this sample of 129 bipolar patients is 42 and 35% respectively. There was no statistically significant difference in BMI in lithium and non-lithium patients with BD. This was also true for secondary outcomes, including average BMI, BMI classes, and MetS, and as well as exploratory outcomes, such as waist circumference, fasting glucose and diabetes, impaired lipids and dyslipidemia, hypertension, and hypothyroidism. However, the most interesting finding is that the groups differed in the markers of insulin resistance, HbA1C and triglycerides. Despite polypharmacy, patients on lithium showed a lower level of insulin resistance.

In other words, our findings suggest overall similar physical and metabolic comorbidity between lithium-using and non-lithium patients with BD. This offers important insight into the adverse effects of ‘real-life’ bipolar pharmacotherapy. In this analysis, the majority of patients were treated with conventional mood stabilizers, lithium or valproate, combined with various antidepressants and second-generation antipsychotics. These medications have been studied and compared more extensively as monotherapy; for example, lithium has previously been reported to cause more clinically significant weight gain than valproate, but less than quetiapine [13, 15]. However, the research is largely mixed regarding weight gain and other side effects (ie. MetS and its constituents) of pharmacotherapy combinations. Previous studies are limited by their focus on individual antipsychotics or mood stabilizers and modest sample sizes [23]. Overall, lithium and non-lithium combinations seem to emerge as comparable in this study.

Interestingly, we have also found lithium to be associated with lower markers of insulin resistance, as measured by HbA1c and triglycerides respectively. This suggests that lithium may confer some protective effects, even in patients treated with multiple psychotropic medications. This finding fits into a larger discussion in the literature that conceptualizes bipolar disorder as being comprised of multiple subgroups, such as lithium-responders. One interpretation of our finding is that lithium-responders, even prior to lithium exposure, represent a subgroup that is naturally less predisposed to insulin resistance. In fact, a recent retrospective study has found that patients with BD and insulin resistance appear to be associated with more severe clinical features and poor response to lithium [24, 25]. This association can potentially be explained by shared pathophysiological mechanisms between bipolar disorder and glucose dysregulation, including genetic and epigenetic links, mitochondrial dysfunction, and hypothalamic-pituitary-adrenal axis alterations [26]. In particular, glycogen synthase kinase-3 beta (GSK-3b), an essential kinase involved in cell metabolism and survival, has been identified as having significant effects on neuronal plasticity in bipolar disorder patients. It may also suppress two important targets of insulin action, glycogen synthase and insulin receptor substrate-1 [27]. Lithium, moreso than other mood stabilizers such as valproate, appears to act as an inhibitor of the GSK-3b enzyme. Therefore, a second and competing hypothesis is that the lithium itself modulates both affective and dysmetabolic disorders [28]. For example, sleep deprivation can rapidly result in insulin resistance, and while in general patients with BD have disrupted circadian rhythms, lithium seems to be more likely to regulate the rhythms [29]. In sum, these pathophysiological explanations are consistent with our results in lithium-responsive bipolar patients, who experienced lower levels of glycosylated hemoglobin and triglycerides.

From a population health perspective, our findings contribute to a growing understanding about metabolic comorbidities in severe mental illness. The prevalence of obesity in this bipolar study group was almost double that of the general Canadian adult population (42.3% versus 24.1%) [30], and almost triple that of the general Québecois adult population (42.3% versus 16.4%) [31]. The same can be said about MetS (35.7% compared with 19.1%) [32]. These Canadian obesity and metabolic syndrome estimates are based on data from the Canadian Health Measures Survey, a national bio-monitoring dataset of adults aged 18–79 (median age 39) and containing approximately equal proportions of sexes (49.6% male). The Québecois obesity estimate derives from a sample similar in demographics (median age 41, 49.5% male) [33, 34]. This confirms previous findings that obesity is startlingly common in bipolar patients and necessitates urgent attention. Prevention and early intervention with lifestyle and pharmacological options is recommended [35] as dysmetabolism has even been reported in patients newly diagnosed with BD [36]. Further, the high concurrence between psychiatric and medical comorbidity argues for primary care models that allow an integrated treatment approach to optimize outcomes.

### Strengths and limitations

This study has several strengths. To begin, the study design is representative of the “real-world”: the enrolment of both bipolar I and II patients, enrolment of patients of all ages, and the inclusion of patients with concomitant psychiatric diagnoses (eg. personality disorder, substance use). The findings can be generalized to tertiary care bipolar outpatients. The grand majority of patients were based at the same hospital and so their lab tests were processed by the same laboratory. Additionally, our sample size was moderate, however, reasonable to compare associations of common somatic comorbidities in patients with BD.

We recognize our study had some limitations. The largest limitation is that the study design was cross-sectional, which makes causation impossible to infer. Furthermore, though common in clinical practice, results should be interpreted with caution as we investigated the association of lithium use in patients with BD treated concurrently with multiple psychotropic medications. We did not collect lithium duration data for the patients in our study; however, based on previous analyses, the average duration of lithium use in our clinic’s lithium users is approximately 10 years [37]. As such, we are confident that our study nonetheless captures chronic lithium users. Body mass index, our primary outcome, is frequently criticized in the literature for its true clinical relevance as it has arbitrarily defined categories, does not differentiate between fat and muscle mass [38], and has been paradoxically associated with lower mortality [39], although the inclusion of waist circumference as a surrogate for obesity helps to mitigate this. Finally, there was an important reliance on self-reported data for non-lab values, such as hypertension. Future studies could (a) incorporate a larger number of patients in a prospective study design to improve statistical power and better quantify effects of lithium (and other mood stabilizers) on metabolic outcome, (b) examine combination treatments more exhaustively, and (c) explore the postulated pathophysiological mechanisms linking bipolar disorders and metabolic dysregulation.

## Conclusion

There is a higher prevalence of obesity and MetS in this sample of patients with BD, when compared to the general population. However, this did not appear to be associated with lithium use, when compared to non-lithium users (e.g. those on anticonvulsants, second-generation antipsychotics). Constituents of MetS also did not significantly differ between lithium-users versus non-users. An important exception was insulin resistance as reflected in HbA1c and triglycerides, with healthier levels in lithium users compared to non-lithium users. Future prospective studies with larger sample sizes will be necessary to confirm whether this association is due to a causal effect of lithium or other factors. A proactive approach focused on prevention, early intervention, and consistent monitoring may help prevent long-term health consequences of obesity.

## Data Availability

The datasets used and/or analyzed during the current study are available from the corresponding author on reasonable request.

## Abbreviations

BD: Bipolar disorder
MetS: Metabolic syndrome
BMI: Body mass index
RCT: Randomized controlled trial
DI: Diabetes insipidus;
MADRS: Montgomery-Asberg Depression Rating Scale
YMRS: Young Mania Rating Scale
MUHC: McGill University Healthcare Center
DMHUI: Douglas Mental Health University Institute
JGH: Jewish General Hospital
NIH: National Institute of Health
NCEP III: National Cholesterol Education Treatment Program III
WHO: World Health Organization
HDL: High-density lipoprotein
HbA1c: Hemoglobin A1c
TSH: Thyroid stimulating hormone
GSK-3b: Glycogen synthase kinase 3-beta
